# A latent profile analysis of post-traumatic growth in spouses of breast cancer patients

**DOI:** 10.3389/fpubh.2025.1634910

**Published:** 2025-08-04

**Authors:** Xiaocui Duan, Xiumu Yang, Yongli Duan, Zhengfu Shen, Yunbo Xiong, Caixia Sun, Yongxia Chen, Yujiao Shao, Xuejun Xu, Shuang Zhao, Zeyu Zhang, Shiqing Zhang, Yue Yang

**Affiliations:** ^1^School of Nursing, Bengbu Medical University, Bengbu, China; ^2^General Practice Education and Development Center, Bengbu Medical University, Bengbu, China; ^3^Fuyang First People’s Hospital, Fuyang, China; ^4^School of Marxism, Bengbu Medical University, Bengbu, China; ^5^The First Affiliated Hospital of Bengbu Medical University, Bengbu, China; ^6^The First People's Hospital of Bengbu, Bengbu, China; ^7^No. 902 Hospital of the People’s Liberation Army Joint Logistics and Security Force, Bengbu, China

**Keywords:** breast cancer, patients, spouses, post-traumatic growth, latent profile analysis

## Abstract

**Introduction:**

Post-traumatic growth is the positive psychological changes that an individual may undergo after coping with traumatic events. It can help individuals alleviate negative emotions, reduce physical and mental stress, and enhance self-efficacy. This study aims to characterize the latent profiles of spouses’ post-traumatic growth and to explore the related factors for each subgroup based on the ABC-X model.

**Methods:**

210 breast cancer couples participated in the survey through convenience sampling and completed the General Information Questionnaire, Post-Traumatic Growth Inventory, Dyadic Coping Inventory, and Resilience Scales.

**Results:**

Latent profile analysis revealed three distinct post-traumatic growth profiles among breast cancer patients’ spouses: low growth-emerging edge group (30.9%), medium growth-moderate progress group (48.7%), and high growth-transcending self group (20.3%). Multivariate logistic regression showed that factors influencing post-traumatic growth in spouses with breast cancer include type of place of residence, resilience, negative coping styles, the number of chemotherapy treatments for the patient, and cancer stage.

**Conclusion:**

There is significant heterogeneity in post-traumatic growth among spouses. Future research could utilize precise psychological interventions to improve post-traumatic growth based on the diverse psychological profiles of spouses, thereby enhancing the mental health of the spousal population.

## 1 Introduction

Currently, breast cancer (BC) ranks as the leading malignancy encountered by women, posing a severe threat to their physical and mental health. Data show that BC accounts for 11.7% of new cancer cases globally (2.26 million out of 19.29 million). BC accounts for 9.2% (420,000) of the 4.57 million new cancer diagnoses in China, making China as the nation with the highest incidence of breast cancer (1). For BC patients, the whole process, from the early diagnosis of the disease to the subsequent radiotherapy, undoubtedly has tremendous physical and mental impacts, severely impairing their healthy lives (2). The family systems theory states that the members of a family interact through emotion, cognition and behavior to form a dynamic network system in which any one member’s behavioral change can ripple through to affect others and the overall family structure (3). Couples are mutually dependent on each other as the emotional family line; therefore, the suffering caused by the disease and treatment suffered by the patient not only reduces the quality of life of the spouses but may also bring about psychological disturbances (4). Notably, 60% of BC patients’ primary caregivers are their spouses (5). Spouses participate in the treatment process of the patients not only to provide for a variety of caregiving needs but also to connect emotionally with the patient, providing comfort, hope, and encouragement, becoming advocates for the patient’s healthcare, managers of self-regulation of emotions, and also connecting with other people for emotional and psychological support, which can help to alleviate multiple stressors (6). Approximately 62% of spouses of BC patients experience clinically significant emotional distress compared to other caregivers, severely affecting their well-being and marital quality (7).

As the field of positive psychology continues to expand, researchers have found that individuals are able to use their wisdom to change their self-perceptions when they experience major stressful events (e.g., life adversities), in order to acquire positive outlooks and values, known as post-traumatic growth (PTG) (8). When confronted with abrupt stressful incidents, spouses, acting as the patient’s caregivers, can still gain positive psychological benefits from the caregiving process. This contributes to diminishing the stress of caregiving, alleviating psychological distress, and enhancing self-efficacy (9). PTG is like a soothing agent for the soul, allowing those who have suffered from the baptism of life to find the courage and strength to move forward and rediscover the meaning and value of life. However, current research often assesses PTG through aggregate scale scores, overlooking population heterogeneity. Latent profile analysis (LPA) is a statistical method of data clustering based on individual characteristics, which classifies populations with similar characteristics through the exogenous variables of the study population and calculates the number and proportion of people in each category, which makes it easier to analyze the features and influencing factors of the population’s various profiles later on and to apply targeted interventions using the profiles as a whole (10). Therefore, this method was used in the current study to investigate the latent profiles of PTG within the spouses’ group.

Resilience is the self-recovery ability of an individual after experiencing adversity that helps him or her adapt, overcome difficulties, and continue to move forward in a positive direction (11). Studies have found that higher resilience facilitates psychological recovery and self-growth in patients (12). When an individual and their intimate partner share coping mechanisms and approaches when encountering a stressful circumstance, this is referred to as dyadic coping (DC) (13). Research suggests that negative coping strategies may obstruct PTG, whereas positive DC may enhance relationship satisfaction and improve patients’ or couples’ psychosocial adaptation (14, 15). Although prior studies have examined the associations between resilience, DC, and PTG, it is unclear whether these factors influence spouses’ PTG.

**Figure 1:**
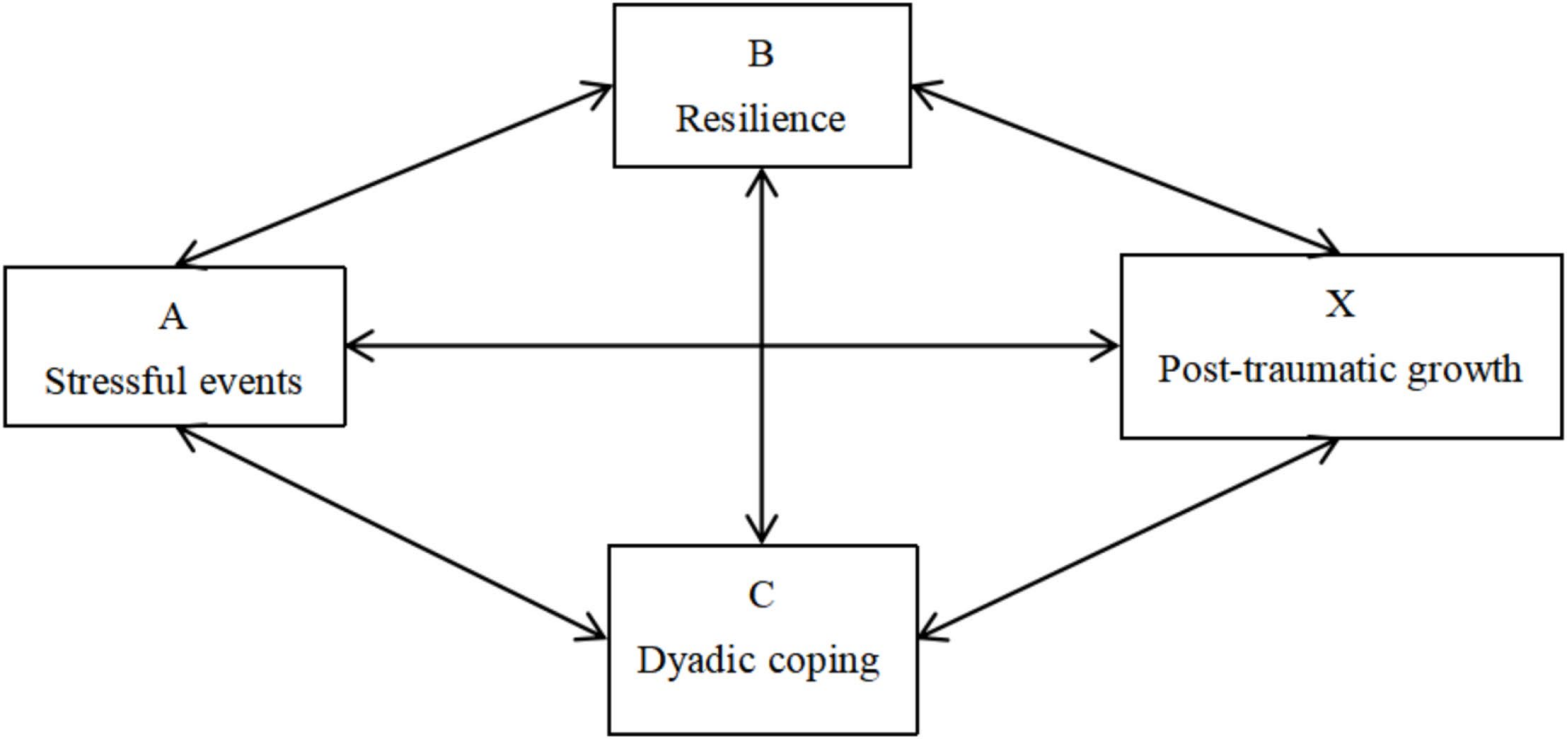
Theoretical framework for post-traumatic growth and related variables.

The ABC-X model is a theoretical framework for analyzing how families cope with stress and consists of four factors: A represents the stressor event that disrupts the family structure; B represents the resources available to the individual experiencing family stress; C represents the person’s opinions or evaluation of going through the traumatic situation; and X represents the outcome of the stress or crisis brought about by the family event (16). Based on this model, we hypothesized that the resources an individual possesses (resilience) and the cognitive appraisal of a stressful event (dyadic coping) predict the effects of events on individuals (post-traumatic growth). Figure 1 presents the theoretical framework of our research. Accordingly, this study aimed to (1) exploring the potential characteristics of PTG among partners of BC patients, and (2) identifying the impact of DC and resilience on PTG across various subgroups of BC patients’ spouses. Exploring these influences could assist healthcare professionals develop strategies to enhance PTG and life quality for spouses of BC patients, offering a benchmark for clinical interventions.

## 2 Methods

### 2.1 Study design and participants

This study utilized a cross-sectional design. From July to December 2023, hospitalized breast cancer patients and their spouses who met the inclusion/exclusion criteria were recruited from the oncology department of a tertiary hospital in Bengbu, China. Inclusion criteria: patients: (1) Female BC patients requiring chemotherapy after surgery; (2) No evidence of recurrence or metastasis; (3) Knowledge of their condition and consent to participate in this research. Spouses: (1) People who are willing to participate in this study, are literate, and have average comprehension abilities; (2) Legal spouse of the patient. Exclusion criteria: (1) Those with mental illness cannot cooperate with this study; (2) No traumatic events in the last 3 months. The sample size involved a consideration of the study variables’ influential factors, multiplied by a factor of 5–10 (17), resulting in an initial estimate of 100–200 participants for the 20 variables under investigation. Ultimately, this study enrolled a final sample size of 210 dyads (patient-spouse pairs).

### 2.2 Recruitment

Participants were initially screened through an electronic medical record system. The screening mainly included demographic characteristics (age, occupation, etc.) and clinical characteristics (cancer stage and surgical manner, etc.). During the patients’ hospitalization, the investigator, assisted by the ward nurse manager, actively communicated with potential participants; this interaction enhanced the trust of participants in the researcher. In the process, the investigator learned about the patients’ perception of the disease and confirmed the spouses’ status as caregivers. The investigator then introduced herself and informed the patients and spouses about the study’s purpose and data collection procedures. The patient-spouse dyads were included in the study only if both the patient and the spouse met the inclusion criteria and provided consent.

### 2.3 Ethical considerations

This study was approved by the Ethics Committee of Bengbu College (Approval No. 2023369). Before data collection, the researcher informed the participants about the purpose and significance of the study, as well as the data confidentiality measures, and explained them that the data will only be used for this research. All participants voluntarily agreed to take part in the research and provided their written consent.

### 2.4 Measures

#### 2.4.1 General information questionnaire

The general information questionnaire collected demographic and clinical characteristics, including: the patient’s educational level, chemotherapy protocol, cancer stage and surgical manner, the spouse’s age, educational level, per capita monthly household income, et al.

#### 2.4.2 Post-traumatic growth inventory (PTGI)

The scale, which Wang et al. (18) translated and localized, was used to assess the beneficial improvements that spouses of patients with BC after experiencing a traumatic event. There are 20 items in 5 dimensions: Personal Strength, Relating to Others, Spiritual Change, Appreciation of Life, New Possibilities, with each entry receiving a rating between 0 and 5, totaling 100 points. Higher scores predict the individual reaps more positive psychological change from the traumatic event. The PTGI scale’s Cronbach’s *α* in our research was 0.856.

#### 2.4.3 Connor and Davidson’s resilience scale (CDRS)

Translated and localized by Yu and Zhang (19), this scale was adopted to evaluate the adaptive capacity of individuals after experiencing a stressful event with BC patients’ spouses. 25 entries in 3 dimensions (Tenacity, Strength, and Optimism), with each entry receiving a rating between 0 and 4, totaling 100 points. The CDRS scale’s Cronbach’s *α* in our research was 0.873.

#### 2.4.4 Dyadic coping inventory (DCI)

Translated and localized by Xu et al. (20) to assess BC couples’ ability to dealing with shock events. This study’s scale, comprising 37 items across 6 dimensions-Stress communication, Supportive coping, Common coping, Delegated coping, Negative coping, Coping evaluation. The DCI scale’s Cronbach’s *α* for spouses in our research was 0.877.

### 2.5 Data collection

Participants were selected according to the study criteria. Questionnaires were distributed with the consent of the other party before the formal survey, and couples completed them separately. If participants had difficulties understanding any items, researchers provided standardized explanations to assist them. All questionnaires were collected immediately upon completion, and participants were thanked for their time.

### 2.6 Statistical analysis

Data from spouses were analyzed using the SPSS 26.0 software. Normally distributed data were characterized by means and standard deviations, while categorical variables were described using frequencies and percentages. For univariate analyses of spouses of BC patients, the Kruskal-Wallis H test was employed to evaluate ordered categorical data, the chi-square test or Fisher’s exact test for group comparisons, and multivariate logistic regression to identify factors potentially influencing PTG categories. Statistical significance was set at *p* < 0.05.

LPA of spousal PTG was conducted using Mplus 7.4 software. Starting with a one-category model, incremental steps were taken until the optimal latent profile emerged. The fit indices for assessing the optimal latent profile were the Akaike Information Criterion (AIC), the Bayesian Information Criterion (BIC), and the sample-size-adjusted BIC (aBIC). A lower value signifies a better model fit. Entropy of information (Entropy), ranges between 0 and 1; a value approaching 1 indicates a higher precision in the classification of the profiles. The Lo–Mendell–Rubin corrected Likelihood Ratio Test (LMR) and Bootstrap-based Likelihood Ratio Test (BLRT), suggest that when *p* < 0.05, the model with the current number of K classifications is superior to the model with K−1 classifications in terms of classification performance (10).

## 3 Results

### 3.1 General information questionnaires

Questionnaires were finally collected from 210 BC couples in this study. The patient’s average age was 47.42 years (SD = 7.825); 69.5% had two or more children; 54.8% had been diagnosed for < 3 months; 57.1% had cancer stage II; 43.8% of the chemotherapy protocol were A/EC; 30.5% received < 2 chemotherapy treatments. The average age of spouses was 48.50 years (SD = 8.320); 53.8% of them lived in towns/urban; 35.7% of families had a per capita monthly income of <3,000 yuan; only 0.9% had a college degree and above.

### 3.2 Subgroups identified by LPA

Through gradual increases of profile categories, five potential profile models were fitted. Table 1 displays the model fit indices for every profile, which shows that when the profiles are 4 and 5, one of the profiles accounted for 0.01, demonstrating no practical significance. In contrast, when the profile is 3, the LMR and BLRT tests are both significant (*p* < 0.05) and entropy > 0.8, and the posterior probability of each profile is 90.7–95.6%, which were greater than 80%. These findings confirm the reliability of the 3 profiles, which was consequently selected as the suitable model.

**Table 1:** Latent profile model fit comparison.

Class	AIC	BIC	aBIC	*P*			
				LMR	BLRT	Entropy	Proportion
1	4950.630	4984.101	4952.415				
2	4674.508	4728.061	4677.364	0.000	0.000	0.857	0.343/0.657
3	4592.697	4666.333	4596.625	0.0001	0.000	0.852	0.314/0.476/0.210
4	4583.123	4676.843	4588.122	0.507	0.013	0.888	0.300/0.210/0.476/0.014
5	4580.134	4693.935	4586.204	0.831	0.364	0.893	0.014/0.295/0.476/0.067/0.147

AIC, Akaike Information Criterion; BIC, Bayesian Information Criterion; aBIC, the sample-size-adjusted BIC; BLRT, Bootstrap-based Likelihood Ratio Test; LMR, Lo-Mendell-Rubin corrected Likelihood Ratio Test.

### 3.3 Characteristics and nomenclature of latent profiles

**Figure 2:**
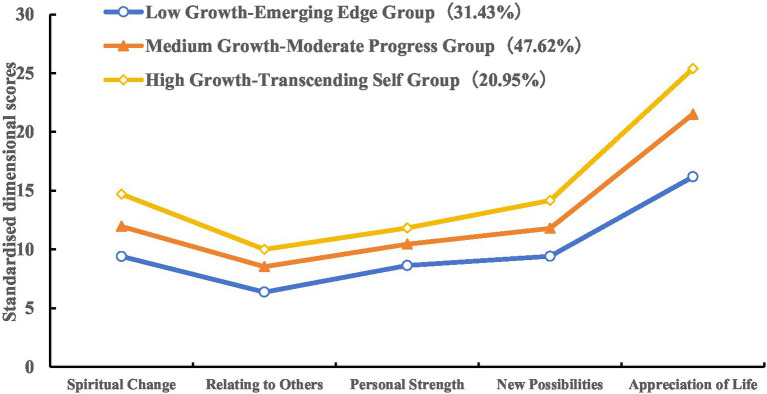
The characteristic distribution of three latent profiles of post-traumatic growth in spouses of breast cancer patients.

Figure 2 depicts the average scores across the five dimensions of PTG for the three profiles. Profile 1 demonstrated consistently lower dimension scores than the other two profiles. Indicating participants were in early growth stages, thus being named “Low Growth-Emerging Edge Group,” accounting for 31.43% (66/210); Profile 2 showed intermediate scores with dimension means slightly above average, reflecting that these individuals have adapted to the changes and are growing in a smooth and sustained manner, hence being named “Medium Growth-Moderate Progress Group,” accounting for 47.62% (100/210); Profile 3 exhibited the highest scores overall and “Relating to Others group” has a higher mean value than the average value of the dimensions. Its dimension scores were higher than those of the other two groups, and “Appreciation of Life” dimension score was significantly higher than those of the other dimensions, so it is named “High Growth-Transcending Self Group,” accounting for 20.95% (44/210).

### 3.4 Univariate analysis of PTG

The univariate analysis showed statistically significant differences (*p* < 0.05) among the three PTG profiles of spouses, particularly regarding several crucial factors: place of residence, monthly household income per capita, cancer stage at diagnosis, the number of chemotherapy treatments, the chemotherapy treatment protocol, resilience levels, and DC strategies. These findings are detailed in Table 2 highlighting the heterogeneity in the PTG experiences of spouses.

**Table 2:** Univariate analysis of post-traumatic growth in spouses of breast cancer patients (*n* = 210).

Variables	Low growth-emerging edge group	Medium growth-moderate progress group	High growth-transcending self group	Test statistics	*P*
Age (years)					
<30	0	1 (1.0)	2 (4.5)	5.153^b^	0.076
30–45	17 (25.8)	26 (26.0)	17 (38.6)		
46–60	44 (66.7)	64 (64.0)	23 (52.3)		
>60	5 (7.6)	9 (9.0)	2 (4.5)		
Education background					
Primary school and below	25 (37.9)	42 (42.0)	13 (29.5)	5.504^b^	0.064
Intermediate	22 (33.3)	42 (42.0)	15 (34.1)		
High or vocational school	15 (22.7)	9 (9.0)	8 (18.2)		
College and above	4 (6.1)	7 (7.0)	8 (18.2)		
Family residence					
Rural	37 (56.1)	50 (50.0)	10 (22.7)	12.917^a^	0.002^*^
Towns/Urban	29 (43.9)	50 (50.0)	34 (77.3)		
Occupational status					
Employed	33 (50.0)	50 (50.0)	28 (63.6)	6.874^d^	0.137
Unemployed/other	28 (42.4)	39 (39.0)	16 (36.4)		
Retired	5 (7.6)	11 (11.0)	0		
Per capita monthly family income (RMB)					
<3,000	29 (43.9)	33 (33.0)	13 (29.5)	6.796^b^	0.033^*^
3,001–4,000	24 (36.4)	40 (40.0)	11 (25.0)		
4,001–5,000	6 (9.1)	20 (20.0)	9 (20.5)		
>5,000	7 (10.6)	7 (7.0)	11 (25.0)		
Cancer stage					
I	16 (24.2)	21 (21.0)	26 (59.1)	18.573^b^	0.001^*^
II	38 (57.6)	67 (67.0)	15 (34.1)		
III	12 (18.2)	12 (12.0)	3 (6.8)		
Number of chemotherapy treatments					
<2	26 (39.4)	33 (33.0)	5 (11.4)	14.451^b^	0.001^*^
2–3	19 (28.8)	30 (30.0)	13 (29.5)		
4–5	13 (19.7)	22 (22.0)	11 (25.0)		
6–8	8 (12.1)	15 (15.0)	15 (34.1)		
Breast-conserving surgery	13 (19.7)	20 (20.0)	8 (18.2)	2.758^d^	0.861
Modified radical surgery	42 (63.6)	56 (56.0)	25 (56.8)		
Simple mastectomy	10 (15.2)	22 (22.0)	9 (20.5)		
Prosthesis implantation	1 (1.5)	2 (2.0)	2 (4.5)		
Number of children					
1	16 (24.2)	29 (29.0)	19 (43.2)	4.643^b^	0.098
≥2	50 (75.8)	71 (71.0)	25 (56.8)		
Diagnosis time					
< 3 months	37 (56.1)	58 (58.0)	20 (45.5)	2.477^b^	0.290
3–6 months	26 (39.4)	32 (32.0)	18 (40.9)		
7–12 months	3 (4.5)	10 (10.0)	4 (9.1)		
> 12 months	0	0	2 (0.4)		
Chemotherapy protocol					
A/EC	28 (42.4)	51 (51.0)	13 (29.5)	18.448^d^	0.012^*^
TEC	1 (1.5)	3 (3.0)	1 (2.3)		
AC-T	14 (21.2)	22 (22.0)	13 (29.5)		
AC-TH/HP	8 (12.1)	9 (9.0)	14 (31.8)		
Unknown	15 (22.7)	15 (15.0)	3 (6.8)		
Resilience	61.80 ± 11.51	63.32 ± 10.71	69.00 ± 12.50	5.679^c^	0.004*
Optimism	8.34 ± 2.27	8.24 ± 2.27	9.34 ± 2.38	3.743^c^	0.025*
Strength	18.73 ± 4.61	19.00 ± 4.33	20.91 ± 5.16	3.415^c^	0.035*
Tenacity	34.73 ± 6.40	36.08 ± 6.27	38.75 ± 7.28	5.045^c^	0.007*
Dyadic coping	113.71 ± 12.09	116.10 ± 13.25	120.30 ± 12.27	3.560^c^	0.030*
Supportive coping	32.23 ± 5.08	32.650 ± 4.986	34.25 ± 4.62	2.371^c^	0.096
Stress communication	23.81 ± 3.87	24.450 ± 3.751	24.86 ± 3.70	0.955^c^	0.387
Negative coping	27.30 ± 3.91	28.39 ± 4.57	30.43 ± 4.10	7.107^c^	0.001^*^
Common coping	16.27 ± 3.11	16.71 ± 2.95	16.82 ± 2.64	0.600^c^	0.550
Delegated coping	14.03 ± 2.01	13.90 ± 1.86	13.93 ± 1.65	0.099^c^	0.906

A/EC, Doxorubicin/Epirubicin, Cyclophosphamide; TEC, Docetaxel, Epirubicin, Cyclophosphamide. AC-T, Doxorubicin, Cyclophosphamide, Paclitaxel. AC-TH/HP, Doxorubicin, Cyclophosphamide, Docetaxel/Paclitaxel, Trastuzumab/Trastuzumab, Pertuzumab.

a χ^2^-value.

b H-value.

c F-value.

d Fisher’s exact test.

### 3.5 Multifactor analyses of three latent profile

Multivariate logistic regression analyses were conducted on the three latent profiles of PTG among BC patients’ spouses, using variables significant in the univariate serving as predictors and the latent profile of PTG of spouses as outcome variables, to explore the determinants of spousal PTG. See Table 3.

**Table 3:** Multivariate regression analysis of three latent profile.

Variables		Profiles 1 versus Profiles 3				Profiles 2 versus Profiles 3			
		*β*	*P*	*OR*	95% *CI*	*β*	*P*	*OR*	95% *CI*
Constant term		6.314	0.012			4.201	0.075		
Resilience		−0.042	0.048	0.959	0.920–1.000	−0.041	0.038	0.960	0.923–0.998
Negative coping		−0.120	0.440	0.887	0.790–0.997	−0.061	0.272	0.941	0.844–1.049
Family residence	Rural	1.091	0.037	2.978	1.071–8.283	1.018	0.040	2.769	1.048–7.317
Number of chemotherapy treatments	<2	1.901	0.029	6.691	1.216–36.820	1.629	0.048	5.099	1.012–25.689
	2–3	0.887	0.204	2.429	0.617–9.559	0.543	0.400	1.721	0.487–6.091
	4–5	0.968	0.183	2.634	0.634–10.951	0.852	0.199	2.344	0.640–8.588
Cancer stage	I	−1.810	0.032	0.164	0.031–0.853	−1.648	0.044	0.192	0.039–0.957
	II	0.212	0.796	1.236	0.248–6.161	0.638	0.426	1.893	0.394–9.104

Profiles 1, Low growth-emerging edge group; Profiles 2, Medium growth-moderate progress group; Profiles 3, High growth-transcending self group.

## 4 Discussion

### 4.1 Three latent profiles exist for PTG in BC patients’ spouses

Our study identified three distinct latent profiles of PTG in BC patients’ spouses using the LPA approach. The model fit indices confirmed significant interindividual variability in spouses’ PTG manifestations. The low growth-emerging edge group, at 31.43%, scored, lower than the other two groups on the overall dimensions of the three profiles, and the “Relating to Others” dimension was significantly lower than the other dimensions. Possible reasons are the low per capita income of the families in this group; the treatment of BC is characterized as continuous, expensive, cyclical and long-term, and good family economic income is a protective factor for the PTG of the spouses. Families with a low income have difficulty coping with the enormous economic pressure and psychological burden brought about by the cost of the treatment (21, 22), which leads to difficulties in generating positive psychological changes in the spouses. In addition, the “Relating to Others” group is a valuable resource for individuals to obtain social support. In establishing relationships with others, individuals not only gain emotional, informational, and social support but also enhance their ability to cope with challenges and grow psychologically (23). Therefore, medical personnel should choose appropriate treatment plans in clinical practice according to the family’s financial situation, and for particularly poor families, social financial assistance can be provided through channels such as the Water Drop Fundraising to alleviate the urgent need for disease treatment and the patients’ worries about seeing a doctor. The medium growth-moderate progress group, comprising 47.62%, is the most populous among the three profiles, which may be that the families in this group have middle income, their spouses are mainly middle-aged people with middle school education, and their families and careers are stable. Even though they may feel fearful during the process of fighting cancer, the middle income of the family, the national health insurance policy for the benefit of the people, the inclusion of biological products in the list of medicines for health insurance, and the execution of the ‘dual-channel’ health insurance reimbursement mechanism have all helped to alleviate their financial pressure. These factors have enabled families to provide better diagnostic protocols and care for their patients, reducing fear among spouses and increasing motivation for treatment (24). The high growth-transcending self group, which accounted for 20.95%, showed the greatest extent of post-traumatic growth among the three subgroups. This group outperformed the others with a much better score on the “Appreciation of Life” dimension. This could be because, in comparison to the other profiles, this one is younger and better educated, which could be explained by the reason that this group is younger and has a higher education level, which helps to improve their cognitive ability and health information literacy. They can have more comprehensive access to disease information and cognitive experiences, broader access to information about the disease, emotional support, and cognitive experiences so that the spouses in this group achieve higher PTG for the spouses as they support patients in their battle against cancer (25–27).

### 4.2 Factors influencing LPA

Rural residence spouses of BC patients who live in the “low growth-emerging edge group” are 1.091 times more numerous than those in the “high growth-transcending self group.” The outcomes are similar to Lu et al.’s (26) study findings. The reasons for this may be, firstly, that in the low growth-emerging edge group, the family income of families living in rural areas is predominantly < 3,000 yuan, and oncology treatment is faced with expensive medical costs, which are unaffordable, exacerbating the spouse’s pessimism and the burden of caregiving, and hampering the emergence of positive psychology (22); Secondly, rural medical and health resources and equipment, as well as access to knowledge of disease popularization activities, are lower than in towns (28). For the treatment of cancer, spouses and patients need to travel to tertiary hospitals, surrounded by unfamiliar people and environments, and the high cost of medical expenditures aggravates the spouse’s fear of the disease, resulting in a reduction in the level of PTG. In addition, in rural areas, the influence of the traditional concept of “cancer,” the labeling of cancer-related illnesses as “incurable,” and the sense of social alienation from those around them can lead to low self-esteem among spouses, which is not conducive to PTG (29). Therefore, we can establish an effective social support system to give psychological support to spouses, such as appealing to friends and relatives to give material or spiritual support to the patient’s family. The government can give appropriate subsidies according to the actual situation of the family of the cancer patient. To better comprehend the psychological condition of their wives and enable them to feel supported by society in their duty as caregivers, medical personnel should communicate more effectively with them. Offer appropriate psychological treatment. Encourage spouses to participate in lectures on disease knowledge in the department to make up for the blind spot of disease knowledge, and at the same time, promote the continuation of care service of Internet^+^ for them to change the cognition of the disease and elevate the degree of PTG.

The patient’s number of chemotherapy treatments was <2. Patients’ spouses had a higher probability of being classified in the “low growth-emerging edge group” (*OR* = 6.691, *p* = 0.029). In agreement with the findings of Lafuenti et al. (30) Some of the patients in this study with <2 chemotherapy treatments were in the post-surgical stage, when the chemotherapy protocol was unknown, and the diagnosis of BC and the absence of breasts subjected the patients to double physical and psychological harm, with the postoperative limb inconvenience, the lack of knowledge related to subsequent chemotherapy, and the prognosis of the disease exacerbating the patients’ psychological stress (31). In the face of sudden events that break the routine and orderly life, the spouse needs to give more care and comfort to the patient, as well as adapt to the change of family roles, which will undoubtedly produce negative psychology in the spouse and hinder the generation of his PTG. With the increase in the number of chemotherapy treatments, the spouse’s caregiving competency improve, the patient’s self-care ability improves and gradually adapts to the treatment plan, and the spouse experiences his value in caregiving, which is conducive to his PTG.

The lower the spouse’s negative coping score, the higher the probability of belonging to the “low growth-emerging edge group.” In agreement with the study of Suo et al. (15). Spouses play many roles in the patient’s disease treatment and recovery process: emotional supporters, family caregivers, financial supporters, decision-making participants, et al. Multiple pressures will undoubtedly produce problems affecting their health, such as sleep disorders, psychological pain, anxiety, et al., and spouses will use avoidance, concealment, and other negative coping styles to divert cancer-related communication and alleviate the patient’s fear of the disease. However, these protective buffering behaviors by spouses are not only detrimental to the generation of their PTG but also lead to less communication and intimacy in couples, which exacerbates the patient’s anxieties about their condition (15, 32). Therefore, medical personnel focus on the patient’s disease treatment at the same time and cannot ignore the psychological attention of the spouse. It should be regarded as husband and wife working together to cope with the pressure of the community to correct the patient’s or spouse’s negative coping and to enhance the positive DC, such as communication and support for each other so as to promote the two sides of the positive psychological changes and the adaptation to the disease.

The likelihood of spouses with cancer stage I patients being categorized under the “high growth-transcending self group” is higher in this research, similar to the findings of Liu et al. (33). Cancer staging assesses the severity of a tumor’s malignancy and the extent of its spread, and the prognosis and survival of patients can be predicted by tumor staging. Patients in the early clinical stage are characterized by mild disease, good prognosis, and long survival, and their spouses have higher hope and confidence in treatment (34). The later the clinical stage, the more likely it is that complications such as metastasis will occur, the patient’s survival rate will be low, treatment options will be limited, and the spouse’s sense of helplessness and hopelessness will increase dramatically, which is not conducive to positive psychology (33). Therefore, medical personnel should focus on the psychology of spouses of late-stage patients and give targeted psychological interventions to alleviate their psychological pain. At the same time, the government, communities, and townships should raise awareness of disease prevention and cancer screening, expand the coverage of the beneficiary population of free health checkups, and increase financial support for health education. Encouraging individuals with financial means to undergo annual routine medical checkups and raising public awareness of self-health will help increase the rate of early diagnosis of diseases, which is crucial for improving the effectiveness of treatments and the overall prognosis for patients.

Spouses with higher resilience showed significantly greater likelihood of belonging to the “high growth-transcending self group,” similar to the study results of Qin et al. (35). Resilience refers to the self-recovery and adaptive capacity of an individual who encounters adversity and shock events by mobilizing his or her internal and external resources and tapping into intrinsic cognitive and psychological attributes (36). Research has pointed out that resilience is a facilitator of PTG; when facing traumatic experiences, couples with strong resilience exhibit good acceptance and resilience, actively mobilize their intrinsic psychological traits, and make full use of external resources to make self-adjustment to adapt to the state of the disease, reduce the psychological burden, and improve their resilience, which facilitates the enhancement of PTG (35). Therefore, medical staff can help elevate PTG by reducing the spouse’s sense of shame, increasing his confidence in the caregiving process, and improving his ability to adapt to the illness through peer support exchange sessions, psychological intervention methods, and information support.

### 4.3 Limitations

Our study’s limitations include: Firstly, the causal relationship between the independent and dependent variables is not yet clear because the design of this study is a cross-sectional type. Future studies should utilize longitudinal tracking to examine the possible causal relationships between these variables. Secondly, the participants were only from the spouses of breast cancer patients in one hospital in Bengbu, China, which limited the external validity of the research results. Multi-center and large-sample studies can be conducted in the future to verify these findings. Additionally, the present study, with only spouses, neglected to consider the interactions of PTG between couples; therefore, in the future, the heterogeneity of different profiles of PTG could be explored for couples as a whole, providing a theoretical basis for the development of family-centred dyadic interventions.

## 5 Conclusion

In this research, through LPA, three latent profiles of PTG were identified in BC patients’ spouses, with differences in the distribution of resilience, coping styles, cancer stage, and the number of chemotherapy treatments across profiles. In the future, healthcare professionals can use resilience as a target for intervention, identify different types of spouses based on influencing factors, and further explore differentiated intervention options to enhance their resilience and standard of living and improve PTG chemotherapy treatments across profiles.

## Data Availability

The original contributions presented in the study are included in the article/supplementary material, further inquiries can be directed to the corresponding author.
